# Incidence of skin and soft-tissue infections in England: 11-year retrospective study

**DOI:** 10.1017/S0950268826101666

**Published:** 2026-05-26

**Authors:** Venanzio Vella, Dominique Derreumaux, Emmanuel Aris, Sachi Mehra, Michele Pellegrini, Mario Contorni, Michael Scherbakov, Fabio Bagnoli

**Affiliations:** 1 Vaccine Epidemiology – Bacterial, GSK, Siena, Italy; 2 Real World Analytics, GSK, Wavre, Belgium; 3 Real World Data Management and Programming, GSK, Bengaluru, India; 4 Vaccines Clinical Sciences, GSK, Siena, Italy; 5 Early Bacterial Vaccine Program, GSK, Siena, Italy; 6 Global Medical Affairs, GSK, Wavre, Belgium; 7 Infectious Diseases RU, GSK, Siena, Italy

**Keywords:** Skin and soft tissue infections, England, chronic ulcer, incidence, epidemiology

## Abstract

Epidemiological estimates for the incidence of skin and soft-tissue infections (SSTIs) in England are limited. We aimed at evaluating the incidence of SSTIs and associated complications through a retrospective, observational 11-year study (2010–2020). We retrieved data on SSTI and associated complications from Clinical Practice Research Datalink, Hospital Episode Statistics, and the Office for National Statistics for England. More than four million SSTI episodes were identified, which corresponded to an overall incidence of 33.1 per 1000 person-years of observation (PYO). The incidence per 1000 PYO was highest in patients with a previous history of SSTI (109.8), ≥65 years (70.1), and those with comorbidities (69.0). The incidence of abscesses/cellulitis/other SSTIs (25.7) was the most frequent, followed by chronic ulcers (4.7) and surgical site infections (2.8). Of the 0.4 million SSTI hospitalizations, ~62.8% were due to abscesses/cellulitis/other SSTIs. Recurrences in the 12 months after the first SSTI were found in 19.5% of cases. Mortality due to SSTI and related complications varied between 0.4% and 0.5% across years, mostly in hospitalized settings. These results show that SSTIs are responsible for a substantial burden of disease in England and will help clinicians in better understanding the epidemiology of SSTI.

## Plain language summary

### SSTI frequency and complications in England

### What is the context?

- Skin and soft-tissue infections (SSTIs) are among the most common infections, and they may affect almost anyone at some point during their life.
- While many studies have provided information about the incidence of SSTIs in the United States, there is negligible data with respect to the incidence of SSTIs in England. We conducted this study to address this data gap by analysing the CPRD database.

### What is new?

- Although the incidence of SSTIs in England was lower compared with that reported in the United States, the rate of hospitalizations was higher.
- Patients were at higher risk for SSTIs if they were older, had comorbidity, or had experienced an SSTI in the past.
- Complicated SSTIs have been increasing, and this is probably the driver of the increasing rates of hospitalizations.

### What is the impact?

- This study fills an important information gap by providing SSTI incidence and recurrence rates.
- The information in this study will help clinicians to have a better understanding of the burden of disease caused by SSTIs.

## Introduction

Skin and soft-tissue infections (SSTIs) are experienced by almost everyone at some point in their life [1, 2]. SSTIs are caused by *Staphylococcus aureus* (33.5–44.6%), *Pseudomonas aeruginosa* (11.1–12.5%), *Escherichia coli* (7.2–14.0%), *Enterococcus* species (6.1–9.3%), and *Streptococcus* species (2.2–4.7%) [2, 3]. However, these infections can also be due to other bacteria (*Treponema pallidum*) [4, 5], fungi [6, 7], and viruses [8, 9]. Some fungal and viral infections of the skin and soft-tissue are very common and disabling (e.g. herpes zoster) [10], and others can be life-threatening (e.g. zygomycosis) [11]. Methicillin-resistant *S. aureus* (MRSA) poses an additional challenge in terms of both treatment and cost [2, 3] and because antimicrobial resistance continues to be one of the greatest threats to society [12]. Because of immunosenescence and age-related comorbidities, the elderlies are at increased risk of acquiring SSTIs [13, 14].

SSTIs are a common reason for visiting a general practitioner (GP), with the most serious SSTI ending up in emergency departments and being admitted to hospitals [15–17]. While 75% of these cases are managed in outpatient settings, SSTIs represent the third most common diagnosis in emergency settings, after chest pain and asthma [15], reflecting the severity of SSTIs [18]. Importantly, the hospital admission rate for SSTIs has increased by 56% in the United Kingdom over the past two decades [13].

While many studies have provided epidemiological estimates for the incidence of SSTIs in the United States [3, 19, 20], there are scant data on the incidence of SSTIs in other countries, including England. To address this gap, we evaluated the incidence of SSTI and associated complications, hospitalizations, and mortality in England between 2010 and 2020. In addition, we assessed the proportion of patients who developed recurrent SSTIs.

## Methods

### Study design and objective

This retrospective, observational study used data from 1 January 2010 to 31 December 2020. The primary objective was to estimate the incidence of SSTI episodes (overall, yearly, by age, sex, comorbidity, type and place of diagnosis, and presence/absence of complications) and the SSTI-associated hospitalization and mortality rates. The secondary objective was to measure the proportion of SSTI episodes by type (e.g. abscess, cellulitis), with and without complications (e.g. lymphadenitis), and by setting (e.g. primary care, outpatient, inpatient). The tertiary objective was to estimate the proportion of individuals who developed recurrent SSTIs following an index episode.

This study complied with all applicable laws regarding individuals’ privacy. No direct contact with patients or primary collection of individual participant data was necessary as we obtained data from the Clinical Practice Research Datalink (CPRD), which collects de-identified data, and only aggregated results are presented. Informed consent, ethics committee, and/or institutional review board approval were not required. However, the study protocol has received approval from CPRD and from an internal GSK protocol review committee.

### Case definitions

SSTI episodes, associated complications, and comorbidities were identified using International Classification of Diseases (ICD) version 10 codes (Supplementary Table S1), and/or CPRD Aurum Medcodeids (Supplementary Table S2).

The earliest recorded date of an SSTI diagnosis was defined as the index SSTI, and any subsequent SSTI was considered part of the same episode providing it occurred within 30 days. The SSTI episodes were divided into the following groups: (a) surgical site infection, (b) chronic ulcers, and (c) abscess/cellulitis/other SSTIs. If several SSTIs were diagnosed during the same episode, the most severe category was assigned using the following hierarchy (in decreasing order of severity): (1) surgical site infection, which includes surgical site infection, infection due to a device or graft, and non-healing surgical wound; (2) chronic ulcers; and (3) abscess/cellulitis/other SSTIs, other SSTIs including mastitis, erysipelas, furuncle/carbuncle, impetigo, folliculitis, infected dermatitis, and unspecified local/subcutaneous/superficial infection.

An SSTI occurrence was considered to have a complication if the complication occurred simultaneously with the infection episode.

The SSTI episodes were treated in either out- or in-patient settings. Episodes that were initially diagnosed in the outpatient setting and which subsequently required hospitalization were regarded as treated in the inpatient setting [20]. Patients presenting to the emergency department and discharged without admission were considered outpatients.

Separate biennial cohorts defined the first recorded episode during the study period, and any subsequent episode was defined as a recurrent episode [21]. The index SSTI was the earliest recorded SSTI in the even years of each biennial cohort. The index SSTI for the measurement of recurrent episodes was defined if a patient was followed up for at least 12 months after the date of the index SSTI. The proportion of index SSTIs with one or more recurrent episodes corresponded to the proportion of the index SSTIs developing one or more SSTIs within a defined follow-up period of 12 months post the index date.

### Data source

SSTI diagnoses were retrieved from the CPRD Aurum database, which collects de-identified patient data from a network of GPs across England. CPRD is jointly sponsored by the UK government’s Medicines and Healthcare products Regulatory Agency (MHRA) and the National Institute for Health Research (NIHR). The database encompasses approximately 60 million patients enrolled over time, of which 18 million registered patients were active as of January 2024. It provides longitudinal data (e.g. for hospitalizations, mortality), with 20 years of follow-up for 25% of patients [22].

Data on SSTI-associated hospitalizations were obtained from the Hospital Episode Statistics (HES), a national database of all patients admitted to the National Health Service (NHS); specifically, we used the HES-admitted patient care and HES outpatient data products.

Data on mortality were retrieved from the Office of National Statistics (ONS) death registry.

### Statistical analysis

Statistical analyses were descriptive in nature and were conducted using SAS System Version 9.4. All incidence rates were estimated, per 1000 person-years of observation (PYO), along with 95% confidence intervals (CIs), by dividing the total number of SSTI episodes during the study period by the person-years contributed by each patient during each calendar year. The 95% confidence intervals were calculated using the Clopper–Pearson method. The contribution of patients to PYO ceased when registration in the CPRD ended, for example, because of death or transfer out. The SSTI incidence was estimated for outpatients and inpatients by age group, gender, and comorbidity. The annual incidence rates were estimated for SSTI episodes (overall, with and without complications), hospitalizations, and deaths.

Proportions of SSTIs by type and associated complications were estimated for outpatients and inpatients. For the entire study period, the number of index cases and proportion of recurrences were also calculated for each biennial cohort. The period of follow-up for recording recurrences was 12 months after the index case, with the last cohort (year 2020) having its period of follow-up in 2021.

## Results

### Incidence of SSTIs

**Figure 1.**
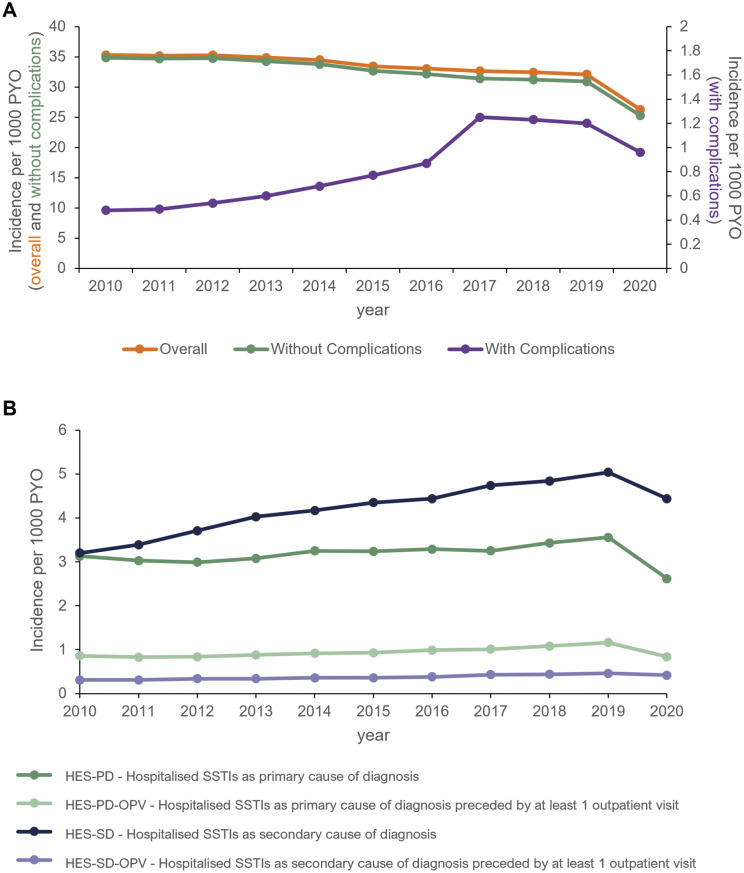
Annual incidence rate per 1000 PYO of SSTIs in England by severity (a) and hospitalization (b). HES-PD, hospitalized with an SSTI as the primary cause of diagnosis; HES-SD, hospitalized with an SSTI as the secondary cause of diagnosis; OPV, outpatient visit; PYO, person-years of observation; SSTI, skin and soft-tissue infection. Figure 1. long description.

More than four million SSTI episodes were identified during the 11-year period covered by our study, with an overall incidence of 33.1 per 1000 PYO (95% CI 33.06, 33.13) (Table 1). Annual incidence rates remained relatively stable, with a slight decline between 2010 and 2019 and a larger decline during 2020, coinciding with the first year of the coronavirus disease 2019 (COVID-19) pandemic. A similar trend was seen for the incidence of SSTIs without complications (Figure 1). Incidence rates were higher in women and those aged 65 years or more (Table 1 **)**.

**Table 1.** Incidence of SSTIs, overall and by risk factors Table 1. long description.

Category	*N* patients	*N* episodes	Days at risk	Incidence rate 1000 PYO (95% CI)
Age group (y)				
<5	163559	189855	2081224277	33.3 (33.2–33.5)
5–17	291756	363297	6536699895	20.3 (20.2–20.4)
18–44	760365	1064433	16307193051	23.8 (23.8–23.9)
45–64	584937	912561	11861826984	28.1 (28.0–28.2)
>65	808331	1521418	7926164318	70.1 (70.0–70.2)
Comorbidities				
Any	967415	1801382	9535853878	69.0 (68.9–69.1)
Previous SSTI	103975	219105	728977904	109.78 (109.3–110.2)
Neoplasm	36209	51416	284325022	66.05 (65.5–66.6)
Diabetes	148680	256266	1499208350	62.43 (62.2–62.7)
HIV	1188	1662	23241753	26.12 (24.9–27.4)
Dermatitis	346208	515367	4436569339	42.43 (42.3–42.5)
Obesity	51165	80199	687881154	42.58 (42.3–42.9)
Chronic kidney disease	5146	8654	30530413	103.53 (101.4–105.7)
Chronic liver disease	832	1309	4561158	104.82 (99.2–110.7)
Peripheral artery and arteriovenous disease	2242	4816	8630926	203.81 (198.1–209.6)
No comorbidity	1631262	2250182	35080210138	23.43 (23.4–23.5)
Gender				
Female	1387556	2226230	22171771432	36.7 (36.6–36.7)
Male	1142304	1825276	22540523268	29.6 (29.5–29.6)
Other	42	58	813825	26.0 (19.8–33.7)
Setting				
GP	2085479	3134703	44713108525	26.6 (25.6–25.6)
HES-PD	330120	388369	44713108525	3.2 (3.2–3.2)
HES-SD	408074	519337	44713108525	4.2 (4.2–4.3)
Outpatient	7379	9155	44713108525	0.1 (0.1–0.1)
Total	2529902	4051564	4471108525	33.1 (33.1–33.1)

CI, confidence interval; GP, general practitioner; HES-PD, hospitalized with an SSTI as the primary cause of diagnosis; HES-SD, hospitalized with an SSTI as the secondary cause of diagnosis; HIV, human immunodeficiency virus; SSTI: skin and soft-tissue infection; PYO, person-years of observation.

**Figure 2.**
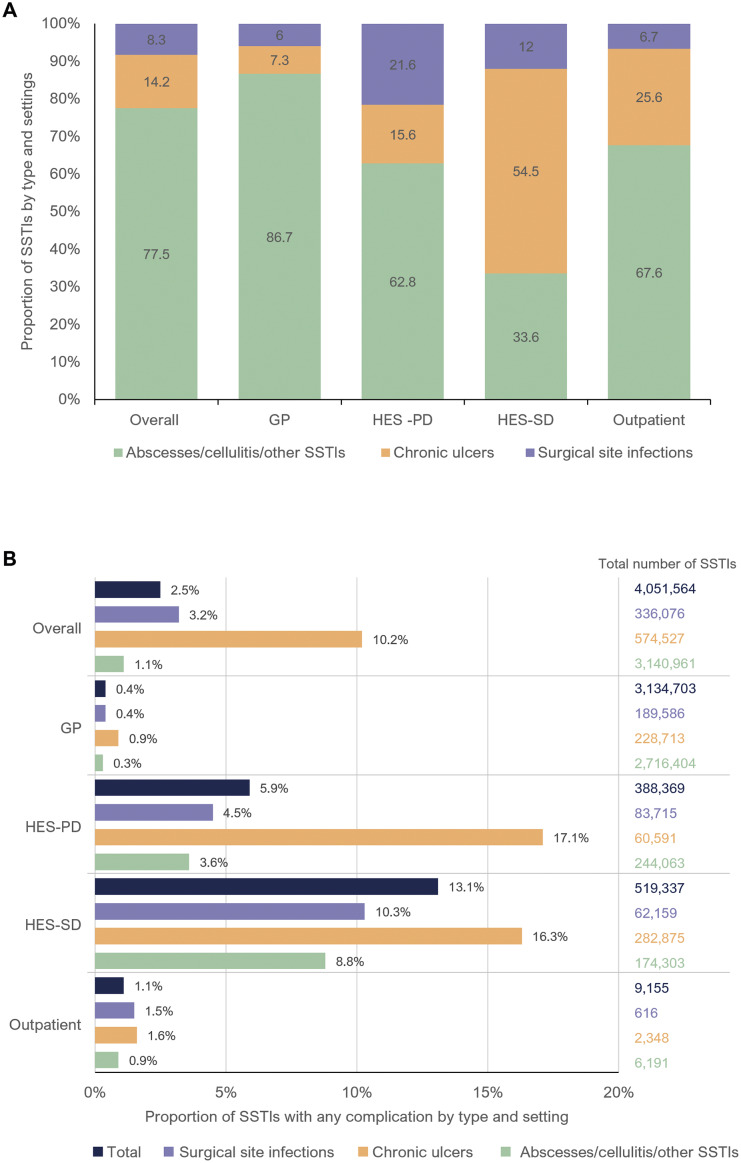
Proportions of SSTIs type by setting (A) and proportion of SSTIs with any complication by type and setting (B). GP, general practitioner; HES-PD, hospitalized with an SSTI as the primary cause of diagnosis; HES-SD, hospitalized with an SSTI as the secondary cause of diagnosis; SSTI, skin and soft-tissue infection. Figure 2. long description.

With regard to the type of SSTI, the incidence and proportion of abscesses/cellulitis/other SSTIs (25.7 per 1000 PYO and 77.5%, respectively) were the most frequent, followed by chronic ulcers (4.7 per 1000 PYO and 14.2%, respectively) and surgical site infections (2.8 per 1000 PYO and 8.3%, respectively), although their predominance varied across settings (Figure 2a). Over the study period, incidence rates of abscess/cellulitis/other SSTIs decreased, from 28.4 to 19.6 per 1000 PYO, whereas the incidence rate of chronic ulcers increased over time, from 4.2 to 4.9 per 1000 PYO. The incidence rates of surgical site infection increased slightly until 2019, from 2.8 to 3.0 per 1000 PYO, and then substantially decreased (1.8 per 1000 PYO during 2020).

### SSTIs with complications

The incidence rate of SSTIs with complications increased, from 0.5 per 1000 PYO during 2010 to 1.0 per 1000 PYO during 2020 (Figure 1a **)**. Among SSTIs with complications (Supplementary Table S3), complicated chronic ulcers increased from 0.2 to 0.6 per 1000 PYO. Complications were associated with 2.5% of SSTI episodes overall (Figure 2b). While only 0.4% of SSTI seen by the GP were complicated, this proportion increased to 1.1% in outpatient units, 5.9% when SSTI was the primary cause of hospitalization, and 13.1% when SSTI was the secondary cause of hospitalization, with chronic ulcer (10.2%) and SSI (3.2%) being most frequently complicated. The most common complications were bacteraemia/endocarditis/sepsis (2.1% of SSTI), followed by osteomyelitis/periostitis/unspecified infection of bones (0.2%) (Table 2).

**Table 2.** Proportion of complications by SSTI type and setting from 2010 to 2020 Table 2. long description.

Setting	SSTI type	*N* of SSTIs	Lymphadenitis (%)	Myositis/necrotizing fasciitis (%)	Gangrene (%)	Osteomyelitis/periostitis /unspecified infection of bone (%)	Bacteraemia/endocarditis/sepsis (%)	Hidradenitis (%)
All	*Abscess/cellulitis/other	3140961	0.01	0.03	0.02	0.05	0.77	0.18
	Chronic ulcer of skin	574527	0.00	0.07	0.27	1.06	9.02	0.01
	**Surgical site infection	336076	0.00	0.07	0.07	0.21	2.87	0.06
	**Total**	**4051564**	**0.01**	**0.04**	**0.06**	**0.21**	**2.12**	**0.15**
GP setting	*Abscess/cellulitis/other	2716404	0.01	0.01	0.01	0.03	0.12	0.16
	Chronic ulcer of skin	228713	0.00	0.01	0.07	0.49	0.28	0.01
	**Surgical site infection	189586	0.00	0.01	0.02	0.08	0.21	0.06
	**Total**	**3134703**	**0.01**	**0.01**	**0.01**	**0.07**	**0.14**	**0.14**
Hospital (SSTI primary diagnosis)	*Abscess/cellulitis/other	244063	0.04	0.09	0.06	0.15	2.78	0.54
	Chronic ulcer of skin	60591	0.01	0.19	0.72	2.63	14.30	0.01
	**Surgical site infection	83715	0.01	0.13	0.13	0.41	3.81	0.11
	**Total**	**388369**	**0.03**	**0.11**	**0.18**	**0.59**	**4.80**	**0.36**
Hospital (SSTI secondary diagnosis)	*Abscess/cellulitis/other	174303	0.03	0.21	0.12	0.22	8.21	0.10
	Chronic ulcer of skin	282875	0.00	0.09	0.32	1.19	15.03	0.01
	**Surgical site infection	62159	0.00	0.19	0.18	0.34	9.75	0.03
	**Total**	**519337**	**0.01**	**0.14**	**0.24**	**0.76**	**12.11**	**0.04**
Outpatient setting	*Abscess/cellulitis/other	6191	0.02	0.05	0.02	0.11	0.26	0.42
	Chronic ulcer of skin	2348	0.00	0.00	0.13	1.19	0.34	0.00
	**Surgical site infection	616	0.00	0.00	0.00	0.81	0.49	0.16
	**Total**	**9155**	**0.01**	**0.03**	**0.04**	**0.44**	**0.29**	**0.29**

GP, general practitioner; *N*, number; SSTI, skin and soft-tissue infection; *Abscess/cellulitis/other = mastitis, cellulitis/abscess, erysipelas, furuncle/carbuncle, impetigo, folliculitis, infected dermatitis and unspecified local/subcutaneous/ superficial infection; **Surgical site infection = surgical site infection/device or graft/non-healing surgical wound.

### Comorbidities

Incidence rates of SSTIs were much higher in patients with comorbidities compared with patients with no comorbidity (69.0 vs. 23.4 per 1000 PYO) with the highest rates in patients with peripheral artery and arteriovenous disease, previous history of SSTI, chronic liver, and kidney disease (Table 1).

### SSTI-associated hospitalizations

Over the study period, 388369 SSTI hospitalizations occurred, with an incidence rate of 3.2 per 1000 PYO. Between 2010 and 2019, the incidence rates for SSTI as primary cause of hospitalization changed slightly from 3.1 to 3.6 per 1000 PYO, while the rates for SSTI as secondary cause of hospitalization increased from 3.2 to 5.0 per 1000 PYO. The incidence rate for an SSTI as a primary or secondary cause of hospitalization decreased during 2020, which coincided with the first year of the COVID-19 pandemic (Figure 1b). The length of hospital stay was almost twice as long when an SSTI was a secondary cause of hospitalization compared with an SSTI as the primary cause of hospitalization (19 vs. 10 days). Abscesses/cellulitis/other SSTIs were the most frequent SSTIs requiring hospitalization (2.0 per 1000 PYO, 95% CI 1.99, 2.00), followed by surgical site infections (0.7 per 1000 PYO, 95% CI 0.7, 0.7) and chronic ulcers (0.5 per 1000 PYO, 95% CI 0.5, 0.5).

### SSTI-associated mortality

**Figure 3.**
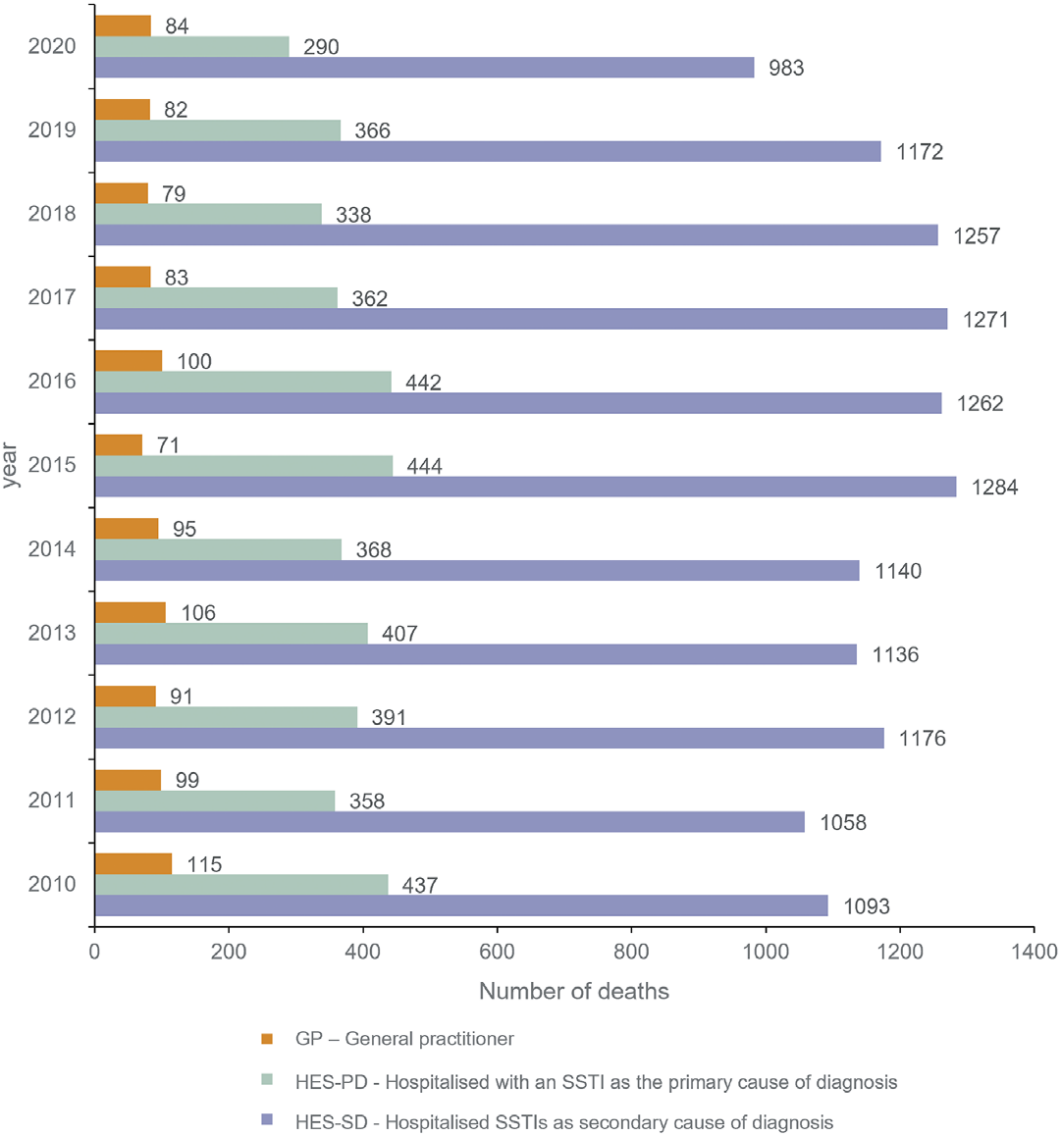
Number of deaths due to SSTIs or related complications by setting*. *Note: Only two deaths were reported in outpatient settings, one in 2013 and one in 2020. HES-PD, hospitalized with an SSTI as the primary cause of diagnosis; HES-SD, hospitalized with an SSTI as the secondary cause of diagnosis; SSTI, skin and soft-tissue infection. Figure 3. long description.

A total of 18168 SSTI-associated deaths occurred during the study period, corresponding to approximately 0.5% of all SSTIs. Most deaths occurred in hospitalized patients, with 4239 and 12911 deaths, respectively, among 388369 and 519337 episodes for which SSTIs were the primary and secondary cause of hospital admission. The mortality was relatively stable, varying from 0.4% to 0.5% between 2010 and 2020. Figure 3 represents the number of deaths due to SSTIs or related complications by setting.

### Recurrent SSTIs

In the biennial cohorts, of the 1625188 index SSTI cases, 19.5% (*N* = 316619) developed a recurrent SSTI episode within 1 year following the index case, corresponding to an overall incidence rate of 219.4 per 1000 PYO. The incidence rate and proportion decreased slightly between 2010 and 2020 (Supplementary Figure S1). Risk factors for recurrences resembled those for all SSTIs, with increased recurrences among those with a previous history of SSTIs, those with comorbidities, and those aged 65 years or more (Supplementary Figure S2).

## Discussion

### Summary

This study provides updated estimates of the incidence of SSTIs, associated hospitalizations, and complications in England.

The presence of complications or risk factors may further worsen the situation by delaying responses to treatment, prolonging hospital stays, and increasing the probability of death. The observed frequency of complications was highest in the inpatient setting, with the most frequent complications being bloodstream infections and osteomyelitis. Risk factors such as age (e.g. 65 years or more), presence of comorbidities (e.g. diabetes, cancer), and a previous history of SSTIs increased the incidence of SSTIs. This is in line with Ray and colleagues who reported that diabetics had twice the risk for SSTI compared with non-diabetics (relative risk: 1.9, 95% CI: 1.90, 1.96) [23]. The observed incidence of SSTIs was the highest in patients with peripheral artery and arteriovenous disease, previous history of SSTI, chronic liver, and kidney disease.

Occasionally, SSTIs can result in mortality. In the present study, the overall mortality due to SSTIs was relatively low and stable, varying from 0.4% to 0.5% between 2010 and 2020. This is in line with the literature [19]. Most fatalities occurred in hospitalized patients with SSTIs as the primary or secondary cause of admission.

Approximately one in five index cases were affected by recurrent episodes. The likelihood of SSTI recurrence increased with age, presence of comorbidities, and previous history of SSTIs. Despite the presence of effective treatment, recurrent SSTIs continue to be quite common, suggesting the needs for more preventive action targeted to patients with risk factors (e.g. age, history of SSTI, comorbidities) that are associated with a high incidence for SSTI and recurrent SSTI [21, 24].

### Strengths and limitations

This study fills a knowledge gap by estimating the burden of SSTI in England. The estimates are robust because the data are drawn from a sizable sample of GP which are the most peripheral outreach post of a universal health care system. It is thus reasonable to assume that the SSTI episodes captured by the GP database provide a fair representation of the SSTI occurring in the population living in the catchment area served by the GP practices included in the CPRD database.

Like any other study, this study also has limitations. Even if the CPRD is one of the largest GP databases of its kind, the enrolled GP might not be fully representative of all the English GP, creating a potential self-selection bias. Another potential issue is that the GP practices do not cover certain populations, such as the homeless, convicts, and patients in long-term care hospices, who are likely to be at higher risk for SSTI.

### Comparison with existing US literature based on insurance claims

The overall annual incidence rate of SSTI episodes, which was relatively stable over the study period (33.1 per 1000 PYO), is somewhat lower than the published estimates for the United States of 34.0–49.6 per 1000 PYO [20, 23, 25]. This could be because SSTI incidence in the present study was based on data from the CPRD database, which holds healthcare records for 25% of the UK population registered with a GP and is known to be representative of the general population [26]. In contrast, the SSTI incidence estimates in the United States were based on insurance claims databases, which might not be fully representative of the US population. Other factors may also have influenced the incidence values between the two countries, such as differences in demography, socioeconomic structure, use of diagnostics, and prevalence of, for example, comorbidities.

In the present study, the incidence of SSTI-associated hospitalizations was higher than previously published estimates. Of the hospitalizations with SSTI as primary cause of admission, 62.8% were cellulitis/abscess/other SSTIs versus the 51.1%–54.5% of SSTI hospitalizations reported in the literature [20, 27]. The management of patients with SSTI-associated hospitalization is resource intensive, with an average expenditure of $22706 per person in the United States [28, 29], bringing into sharp focus the importance of managing SSTIs before they deteriorate into complicated SSTI requiring hospitalization.

### Implication for research

It is evident that the epidemiology of SSTIs in England is changing, especially with respect to advancing age and the presence of comorbidities. Data from this study will be useful for formulating hypotheses for clinical studies aimed at preventing or treating SSTIs. Knowledge of SSTI epidemiology and factors that increase the risk of SSTIs is important for clinicians not only to combat these infections but also to optimize their outcomes.

## Supporting information

10.1017/S0950268826101666.sm001 Vella et al. supplementary material Vella et al. supplementary material

## Data Availability

The data that support the findings of this study are available from CPRD (https://cprd.com/), HES, and ONS data Copyright © (2024), reused with the permission of The Health & Social Care Information Centre. All rights reserved. Restrictions apply to the availability of these data, which were used under licence for the current study and so are not publicly available. This study is based, in part, on data from the Clinical Practice Research Datalink obtained under licence from the UK Medicines and Healthcare products Regulatory Agency. The data are provided by patients and collected by the NHS as part of their care and support. The interpretation and conclusions contained in this study are those of the authors alone.

## Supplementary material

The supplementary material for this article can be found at http://doi.org/10.1017/S0950268826101666.

## Patient and public involvement

Patients and/or the public were not involved in this study. No direct contact with patients or primary collection of individual participant data was necessary, as we obtained data from the Clinical Practice Research Datalink (CPRD).

## Figure 1. Long description

Panel A is a line graph showing incidence rate of SSTIs per 1000 PYO over time, overall, with and without complications. The years, from 2010 to 2020, are shown on the x-axis. The incidence per 1000 PYO is plotted on y-axes. The primary y-axis shows SSTI incidence overall and for uncomplicated SSTI and ranges from 0 to 40 per 1000 PYO. The secondary axis shows the incidence of complicated SSTI and ranges from 0 to 2 per 1000 PYO. Overall SSTI incidence decreases over time, more markedly in 2020. SSTI without complications follows overall SSTI closely. Incidence of SSTIs with complications increases until 2017 and then decreases.

Panel B is a line graph showing incidence rate per 1000 PYO of hospitalized SSTIs over time for hospitalized SSTIs as a primary or a secondary cause of diagnosis, each preceded or not by at least an outpatient visit. The years, from 2010 to 2020, are shown on the x-axis. The incidence is plotted on the y-axis, spanning from 0 to 6 per 1000 PYO. Hospitalized SSTIs as a secondary cause of diagnosis show the highest incidence throughout the years and increase from 2010 to 2019 before decreasing in 2020. Hospitalized SSTIs preceded by at least one outpatient visit have lower incidences. The incidences of hospitalized SSTIs as a primary cause of diagnosis and hospitalized SSTIs as a primary or secondary cause of diagnosis preceded by at least one outpatient visit are relatively stable until 2019, before decreasing.

Navigate back to Figure 1..

## Figure 2. Long description

Panel A is a stacked bar graph depicting the proportion of SSTIs by type and setting. Types include surgical site infection, chronic ulcer of skin, abscess, cellulitis, or other SSTIs. Settings include GP, hospitalized with an SSTI as the primary cause of diagnosis, hospitalized with an SSTI as the secondary cause of diagnosis, outpatient, and overall. For each setting presented on the x-axis, the different SSTI types are stacked on vertical bars, with the sum adding up to a proportion of 100%. In the hospitalized with an SSTI as the secondary cause of diagnosis setting, chronic ulcers are the most frequent SSTI type. In all other settings, abscess, cellulitis, or other SSTIs are the most frequent SSTI type.

Panel B is a bar graph depicting the proportion of SSTIs with any complication by type and setting. The x-axis presents the proportion of SSTI with any complication and ranges from 0%–20%. Settings and SSTI types are as previously stated. Chronic ulcers are the SSTI type that most frequently present complications across settings.

Navigate back to Figure 2..

## Table 2. Long description

This table shows the proportion of complications by SSTI type and setting. SSTI types include surgical site infection, chronic ulcer of skin, and abscess, cellulitis or other SSTIs. Settings include GP, hospitalised with an SSTI as the primary cause of diagnosis, and hospitalised with an SSTI as the secondary cause of diagnosis, outpatient, and overall. Complications include lymphadenitis, myositis or necrotizing fasciitis, gangrene, osteomyelitis or periostitis or unspecified infection of bone, bacteraemia or endocarditis or sepsis, and hidradenitis. Bacteraemia or endocarditis or sepsis is the most frequent complication type in all settings. All complications are more frequent in the hospitalised with an SSTI as the secondary cause of diagnosis setting.

Navigate back to Table 2..

## Figure 3. Long description

This is a bar graph presenting the number of deaths due to SSTIs or related complications by setting and year. The number of deaths is shown on the x-axis, which ranges from 0 to 1400. Years are presented on the y-axis from 2010 to 2020. Settings include GP, hospitalized with an SSTI as the primary cause of diagnosis, and hospitalized with an SSTI as the secondary cause of diagnosis. Throughout the years, most deaths due to SSTIs or related complications occurred in the hospitalized setting, with SSTI as the secondary cause of diagnosis.

Navigate back to Figure 3..

## Table 1. Long description

This table presents the number of patients, of episodes, of days at risk and finally, the incidence rate per 1000 PYO with 95%CIs by age group, gender, comorbidity, and setting, and overall. Age groups include <5 years, 5–17 years, 18–44 years, 45–64 years, and >65 years. Settings include GP, hospitalised with an SSTI as the primary cause of diagnosis, hospitalised with an SSTI as the secondary cause of diagnosis, and outpatient. Comorbidities include previous SSTI, neoplasm, diabetes, HIV, dermatitis, obesity, chronic kidney disease, chronic liver disease, peripheral artery and arteriovenous disease. Incidence is higher in males, in older patients, and in the GP setting. Incidence is also higher in patients with comorbidities, especially in those with peripheral artery and arteriovenous disease, chronic liver disease previous SSTI, or chronic kidney disease.e.

Navigate back to Table 1..

## References

[1] Martinez N (2020) Skin and Soft-Tissue Infections: It’s More Than Just Skin Deep. Advanced Emergency Nursing Journal 42(3), 196–203. 10.1097/TME.0000000000000312. 32739948

[2] Dryden MS (2010) Complicated skin and soft tissue infection. Journal of Antimicrobial Chemotherapy 65(Supplement 3), iii35–iii44. 10.1093/jac/dkq302. 20876627

[3] Moet GJ, et al. (2007) Contemporary causes of skin and soft tissue infections in North America, Latin America, and Europe: report from the SENTRY Antimicrobial Surveillance Program (1998–2004). Diagnostic Microbiology and Infectious Disease 57(1), 7–13. 10.1016/j.diagmicrobio.2006.05.009. 17059876

[4] Ciccarese G, et al. (2024) Atypical Manifestations of Syphilis: A 10-Year Retrospective Study. Journal of Clinical Medicine 13(6), 1603. 10.3390/jcm13061603. 38541829 PMC10971508

[5] Lapenda I, et al. (2026) Histologic Features of Secondary Syphilis: A Systematic Review and Meta-Analysis. The American Journal of Dermatopathology 48(4), 257–266. 10.1097/DAD.0000000000003165. 41849752

[6] Fadlalla S, et al. (2026) Skin signs of invasive fungal diseases: diagnostic clues for clinicians. Current Opinion in Infectious Diseases 39(2), 90–96. 10.1097/QCO.0000000000001180. 41505800

[7] Ma Y, Wang X and Li R (2021) Cutaneous and subcutaneous fungal infections: recent developments on host-fungus interactions. Current Opinion in Microbiology 62, 93–102. 10.1016/j.mib.2021.05.005. 34098513

[8] Goldust M (2023) Viral Diseases in Dermatology. Viruses 15(2), 513. 10.3390/v15020513. 36851727 PMC9965551

[9] Khalil N, et al. (2024) Viral infections in atopic dermatitis. Clinical and Experimental Dermatology 50(1), 46–55. 10.1093/ced/llae304. 39097528

[10] Patil A, Goldust M and Wollina U (2022) Herpes zoster: A Review of Clinical Manifestations and Management. Viruses 14(2), 192. 10.3390/v14020192. 35215786 PMC8876683

[11] Roden MM, et al. (2005) Epidemiology and outcome of zygomycosis: a review of 929 reported cases. Clinical Infectious Diseases 41(5), 634–653. 10.1086/432579. 16080086

[12] European Centre for Disease Prevention and Control. Surveillance of antimicrobial resistance in Europe 2018. Stockholm: ECDC; 2019. Surveillance of antimicrobial resistance in Europe 2018 (europa.eu) https://www.ecdc.europa.eu/en/publications-data/surveillance-antimicrobial-resistance-europe-2018 (accessed 23 January 2024).

[13] Samannodi M (2022) Hospital Admissions Related to Infections and Disorders of the Skin and Subcutaneous Tissue in England and Wales. Healthcare (Basel) 10(10), 2028. 10.3390/healthcare10102028. PMC9601618 36292475

[14] Falcone M and Tiseo G (2023) Skin and soft tissue infections in the elderly. Current Opinion in Infectious Diseases 36(2), 102–108. 10.1097/QCO.0000000000000907. 36718942 PMC10325572

[15] Ki V and Rotstein C (2008) Bacterial skin and soft tissue infections in adults: A review of their epidemiology, pathogenesis, diagnosis, treatment and site of care. Canadian Journal of Infectious Diseases and Medical Microbiology 19(2), 173–184. 10.1155/2008/846453. 19352449 PMC2605859

[16] Schofield JK, et al. (2011) Skin conditions are the commonest new reason people present to general practitioners in England and Wales. British Journal of Dermatology 165(5), 1044–1050. 10.1111/j.1365-2133.2011.10464.x. 21692764

[17] Mistry RD (2020) Skin and Soft Tissue Infections in Ambulatory Care Settings: Setting a New Trend. Clinical Infectious Diseases 70(12), 2719–2720. 10.1093/cid/ciz980. 31605476

[18] Grossi AP, et al. (2022) Skin infections in Europe: a retrospective study of incidence, patient characteristics and practice patterns. International Journal of Antimicrobial Agents 60(3), 106637. 10.1016/j.ijantimicag.2022.106637. 35820533

[19] Kaye KS, et al. (2015) Rising United States Hospital Admissions for Acute Bacterial Skin and Skin Structure Infections: Recent Trends and Economic Impact. PLoS One 10(11), e0143276. 10.1371/journal.pone.0143276. 26599005 PMC4657980

[20] Miller LG, et al. (2015) Incidence of skin and soft tissue infections in ambulatory and inpatient settings, 2005–2010. BMC Infectious Diseases 15(1), 362. 10.1186/s12879-015-1071-0. 26293161 PMC4546168

[21] Vella V, et al. (2021) Staphylococcus aureus Skin and Soft Tissue Infection Recurrence Rates in Outpatients: A Retrospective Database Study at 3 US Medical Centers. Clinical Infectious Diseases 73(5), e1045–e1053. 10.1093/cid/ciaa1717. 33197926 PMC8423503

[22] Clinical Practice Research Datalink. https://cprd.com/ (accessed 23 January 2024).

[23] Ray GT, Suaya JA and Baxter R (2013) Incidence, microbiology, and patient characteristics of skin and soft-tissue infections in a U.S. population: a retrospective population-based study. BMC Infectious Diseases 13(1), 252. 10.1186/1471-2334-13-252. 23721377 PMC3679727

[24] May L, et al. (2017) Incidence and factors associated with emergency department visits for recurrent skin and soft tissue infections in patients in California, 2005–2011. Epidemiology and Infection 145(4), 746–754. 10.1017/S0950268816002855. 27917738 PMC9507778

[25] Casey JA, et al. (2013) A population-based study of the epidemiology and clinical features of methicillin-resistant Staphylococcus aureus infection in Pennsylvania, 2001–2010. Epidemiology and Infection 141(6), 1166–1179. 10.1017/S0950268812001872. 22929058 PMC4360139

[26] Mahadevan P, et al. (2022) Completeness and representativeness of small area socioeconomic data linked with the UK Clinical Practice Research Datalink (CPRD). Journal of Epidemiology and Community Health 76(10), 880–886. 10.1136/jech-2022-219200. 35902219 PMC9484378

[27] Suaya JA, et al. (2013) Skin and soft tissue infections and associated complications among commercially insured patients aged 0–64 years with and without diabetes in the U.S. PLoS One 8(4), e60057. 10.1371/journal.pone.0060057. 23593162 PMC3622669

[28] Lee GC, et al. (2015) Incidence and cost of skin and soft tissue infections in the United States. Value in Health 18(3), A245. 10.1016/j.jval.2015.03.1424.

[29] Nathwani D, Dryden M and Garau J (2016) Early clinical assessment of response to treatment of skin and soft-tissue infections: how can it help clinicians? Perspectives from Europe. International Journal of Antimicrobial Agents 48(2), 127–136. 10.1016/j.ijantimicag.2016.04.023. 27283729

