# Supplementary material for: Incidence of skin and soft-tissue infections in England: 11-year retrospective study
Source: Epidemiol Infect. 2026 May 26;154:e80. doi: 10.1017/S0950268826101666 (PMC13279958; doi:10.1017/S0950268826101666)
Supplement: Vella et al. supplementary material [file S0950268826101666sup001.zip › STAPH_EPI_CPRD database study_Supplement.docx]

**Incidence of skin and soft tissue infections in England: 11-year retrospective study**Venanzio Vella ^1^*, Dominique Derreumaux^2^, Emmanuel Aris^2^, Sachi Mehra^3^, Michele Pellegrini^4^, Mario Contorni^5^, Michael Scherbakov^6^, Fabio Bagnoli^7^

1. Vaccine Epidemiology – Bacterial, GSK, Siena, Italy
2. Real World Analytics, GSK, Wavre, Belgium
3. Real World Data Management and Programming, GSK, Bengaluru, Karnataka, India
4. Vaccines Clinical Sciences, GSK, Siena, Italy
5. Early Bacterial Vaccine Program, GSK, Siena, Italy
6. Global Medical Affairs, GSK, Wavre, Belgium
7. Infectious Diseases RU, GSK, Siena, Italy

**SUPPLEMENT**

**Table S1** ICD-10 codes for clinical diagnosis of SSTIs and associated complications

| **Diagnosis** | **ICD-10 code** |
| --- | --- |
| Surgical site infection | K681, O86.0, O90.2, T81.4, |
| Non-healing surgical wound | T81.8 |
| Infection due to device or graft | T85.7 |
| Chronic ulcer of skin | L89.x, L97.x |
| Abscess or Carbuncle or furuncle | L02.0 to L02.6, L02.8, L02.9, |
| Cellulitis | L03.x |
| Erysipelas | A46 |
| Impetigo | L01.x |
| infected dermatitis | L30.3 |
| Folliculitis | L66.3, L73.8 |
| Mastitis | N61x, O91.0, O91.1 |
| Unspecified local /subcutaneous/ superficial infection | L08.0, L08.8, L08.9, S00.9, S10.9, S40.8, S40.9, S50.8, S50.9, S60.8, S60.9, S70.9, S80.8, S80.9, S90.8, S90.9 |
| Acute lymphadenitis | L04.9 |
| Myositis | M60 |
| Necrotising fasciitis | M72.6 |
| Gangrene | I96 |
| Osteomyelitis | M86.x |
| Bacteraemia | R78.8 |
| Endocarditis | I33.x |
| Sepsis | A40x, A41.x, R65.2 |
| Complication Diagnosis | L73.2 |

ICD-10, International Classification of Diseases version 10 code; SSTIs, skin and soft tissue infections.

**Table S2** Medicodeids for clinical diagnosis of SSTIs, associated complications and comorbidities.

See “Table S2 Medicodeids” excel spreadsheet

Table S3: Incidence rate per 1000 PYO of SSTIs by type, with and without complications

|  | **TOTAL** | | **2010** | **2011** | **2012** | **2013** | **2014** | | | **2015** | **2016** | **2017** | **2018** | | **2019** | **2020** |
| --- | --- | --- | --- | --- | --- | --- | --- | --- | --- | --- | --- | --- | --- | --- | --- | --- |
| N days at risk | 44713108525 | | 3748862932 | 3796671428 | 3869569440 | 3899602145 | 3945852920 | | | 4033835044 | 4152644901 | 4248735684 | 4343089567 | | 4346930984 | 4327313480 |
| **SSTIs overall** | | | | | | | | | | | | | | | | |
| N episodes | 4051564 | 362473 | | 365803 | 373791 | 372316 | | 372494 | 369531 | | 375646 | 380031 | 385830 | 382309 | | 311340 |
| **Surgical Site Infection/Device or graft/Non-healing Surgical Wound** | | | | | | | | | | | | | | | | |
| N episodes | 336076 | | 28412 | 28244 | 28749 | 29104 | 30316 | | | 30652 | 32562 | 34699 | 35935 | | 35898 | 21505 |
| IR | 2.75 | | 2.77 | 2.72 | 2.71 | 2.73 | 2.81 | | | 2.78 | 2.86 | 2.98 | 3.02 | | 3.02 | 1.82 |
| 95% CI LL | 2.74 | | 2.74 | 2.69 | 2.68 | 2.69 | 2.77 | | | 2.74 | 2.83 | 2.95 | 2.99 | | 2.99 | 1.79 |
| 95% CI UL | 2.75 | | 2.8 | 2.75 | 2.75 | 2.76 | 2.84 | | | 2.81 | 2.9 | 3.01 | 3.05 | | 3.05 | 1.84 |
| **Chronic ulcer of skin** | | | | | | | | | | | | | | | | |
| N episodes | 574527 | | 42873 | 43075 | 45986 | 48835 | 50161 | | | 52663 | 54500 | 56984 | 59522 | | 61998 | 57930 |
| IR | 4.69 | | 4.18 | 4.14 | 4.34 | 4.57 | 4.64 | | | 4.77 | 4.79 | 4.9 | 5.01 | | 5.21 | 4.89 |
| 95% CI LL | 4.68 | | 4.14 | 4.1 | 4.3 | 4.53 | 4.6 | | | 4.73 | 4.75 | 4.86 | 4.97 | | 5.17 | 4.85 |
| 95% CI UL | 4.71 | | 4.22 | 4.18 | 4.38 | 4.61 | 4.68 | | | 4.81 | 4.83 | 4.94 | 5.05 | | 5.25 | 4.93 |
| **Abscess/Cellulitis/Erysipelas/Folliculitis/Impetigo/Furuncle & Carbuncle/Mastitis/Other** | | | | | | | | | | | | | | | | |
| N episodes | 3140961 | | 291188 | 294484 | 299056 | 294377 | 292017 | | | 286216 | 288584 | 288348 | 290373 | | 284413 | 231905 |
| IR | 25.66 | | 28.37 | 28.33 | 28.23 | 27.57 | 27.03 | | | 25.92 | 25.38 | 24.79 | 24.42 | | 23.9 | 19.57 |
| 95% CI LL | 25.63 | | 28.27 | 28.23 | 28.13 | 27.47 | 26.93 | | | 25.82 | 25.29 | 24.7 | 24.33 | | 23.81 | 19.49 |
| 95% CI UL | 25.69 | | 28.47 | 28.43 | **28.33** | 27.67 | 27.13 | | | 26.01 | 25.48 | 24.88 | 24.51 | | 23.99 | 19.65 |
| **SSTI with complication** | | | | | | | | | | | | | | | | |
| N episodes | 102567 | 4883 | | 5089 | 5686 | 6446 | | 7298 | | 8451 | 9932 | 14494 | 14646 | 14232 | | 11410 |
| **Surgical Site Infection/Device or graft/Non-healing Surgical Wound** | | | | | | | | | | | | | | | | |
| N episodes | 10868 | | 664 | 644 | 627 | 698 | 732 | | | 862 | 991 | 1566 | 1556 | | 1515 | 1013 |
| IR | 0.09 | | 0.06 | 0.06 | 0.06 | 0.07 | 0.07 | | | 0.08 | 0.09 | 0.13 | 0.13 | | 0.13 | 0.09 |
| 95% CI LL | 0.09 | | 0.06 | 0.06 | 0.05 | 0.06 | 0.06 | | | 0.07 | 0.08 | 0.13 | 0.12 | | 0.12 | 0.08 |
| 95% CI UL | 0.09 | | 0.07 | 0.07 | 0.06 | 0.07 | 0.07 | | | 0.08 | 0.09 | 0.14 | 0.14 | | 0.13 | 0.09 |
| **Chronic ulcer of skin** | | | | | | | | | | | | | | | | |
| N episodes | 58541 | | 2386 | 2539 | 3090 | 3571 | 4066 | | | 4737 | 5639 | 8355 | 8590 | | 8474 | 7094 |
| IR | 0.48 | | 0.23 | 0.24 | 0.29 | 0.33 | 0.38 | | | 0.43 | 0.50 | 0.72 | 0.72 | | 0.71 | 0.60 |
| 95% CI LL | 0.47 | | 0.22 | 0.23 | 0.28 | 0.32 | 0.36 | | | 0.42 | 0.48 | 0.7 | 0.71 | | 0.7 | 0.58 |
| 95% CI UL | 0.48 | | 0.24 | 0.25 | 0.3 | 0.35 | 0.39 | | | 0.44 | 0.51 | 0.73 | 0.74 | | 0.73 | 0.61 |
| **Abscess/Cellulitis/Erysipelas/Folliculitis/Impetigo/Furuncle & Carbuncle/Mastitis/Other** | | | | | | | | | | | | | | | | |
| N episodes | 33158 | | 1833 | 1906 | 1969 | 2177 | 2500 | | | 2852 | 3302 | 4573 | 4500 | | 4243 | 3303 |
| IR | 0.27 | | 0.18 | 0.18 | 0.19 | 0.2 | 0.23 | | | 0.26 | 0.29 | 0.39 | 0.38 | | 0.36 | 0.28 |
| 95% CI LL | 0.27 | | 0.17 | 0.18 | 0.18 | 0.2 | 0.22 | | | 0.25 | 0.28 | 0.38 | 0.37 | | 0.35 | 0.27 |
| 95% CI UL | 0.27 | | 0.19 | 0.19 | 0.19 | 0.21 | 0.24 | | | 0.27 | 0.30 | 0.40 | 0.39 | | 0.37 | 0.29 |
| **SSTI without complication** | | | | | | | | | | | | | | | | |
| N episodes | 3948997 | 357590 | | 360714 | 368105 | 365870 | | 365196 | 361080 | | 365714 | 365537 | 371184 | | 368077 | 299930 |
| **Surgical Site Infection/Device or graft/Non-healing Surgical Wound** | | | | | | | | | | | | | | | | |
| N episodes | 325208 | | 27748 | 27600 | 28122 | 28406 | 29584 | | | 29790 | 31571 | 33133 | 34379 | | 34383 | 20492 |
| IR | 2.66 | | 2.7 | 2.66 | 2.65 | 2.66 | 2.74 | | | 2.7 | 2.78 | 2.85 | 2.89 | | 2.89 | 1.73 |
| 95% CI LL | 2.65 | | 2.67 | 2.62 | 2.62 | 2.63 | 2.71 | | | 2.67 | 2.75 | 2.82 | 2.86 | | 2.86 | 1.71 |
| 95% CI UL | 2.67 | | 2.74 | 2.69 | 2.69 | 2.69 | 2.77 | | | 2.73 | 2.81 | 2.88 | 2.92 | | 2.92 | 1.75 |
| **Chronic ulcer of skin** | | | | | | | | | | | | | | | | |
| N episodes | 515986 | | 40487 | 40536 | 42896 | 45264 | 46095 | | | 47926 | 48861 | 48629 | 50932 | | 53524 | 50836 |
| IR | 4.21 | | 3.94 | 3.9 | 4.05 | 4.24 | 4.27 | | | 4.34 | 4.3 | 4.18 | 4.28 | | 4.5 | 4.29 |
| 95% CI LL | 4.2 | | 3.91 | 3.86 | 4.01 | 4.2 | 4.23 | | | 4.3 | 4.26 | 4.14 | 4.25 | | 4.46 | 4.25 |
| 95% CI UL | 4.23 | | 3.98 | 3.94 | 4.09 | 4.28 | 4.31 | | | 4.38 | 4.34 | 4.22 | 4.32 | | 4.54 | 4.33 |
| **Abscess/Cellulitis/Erysipelas/Folliculitis/Impetigo/Furuncle & Carbuncle/Mastitis/Other** | | | | | | | | | | | | | | | | |
| N episodes | 3107803 | | 289355 | 292578 | 297087 | 292200 | 289517 | | | 283364 | 285282 | 283775 | 285873 | | 280170 | 228602 |
| IR | 25.39 | | 28.19 | 28.15 | 28.04 | 27.37 | 26.8 | | | 25.66 | 25.09 | 24.4 | 24.04 | | 23.54 | 19.3 |
| 95% CI LL | 25.36 | | 28.09 | 28.04 | 27.94 | 27.27 | 26.7 | | | 25.56 | 25 | 24.31 | 23.95 | | 23.45 | 19.22 |
| 95% CI UL | 25.42 | | 28.29 | 28.25 | 28.14 | 27.47 | 26.9 | | | 25.75 | 25.18 | 24.49 | 24.13 | | 23.63 | 19.37 |

N: number; 95% CI LL/UL: 95% confidence interval lower limit/ upper limit; SSTIs: skin and soft tissue infections; PYO, person-years of observation.

**Figure S1** Percentage and incidence of recurrent SSTIs

**
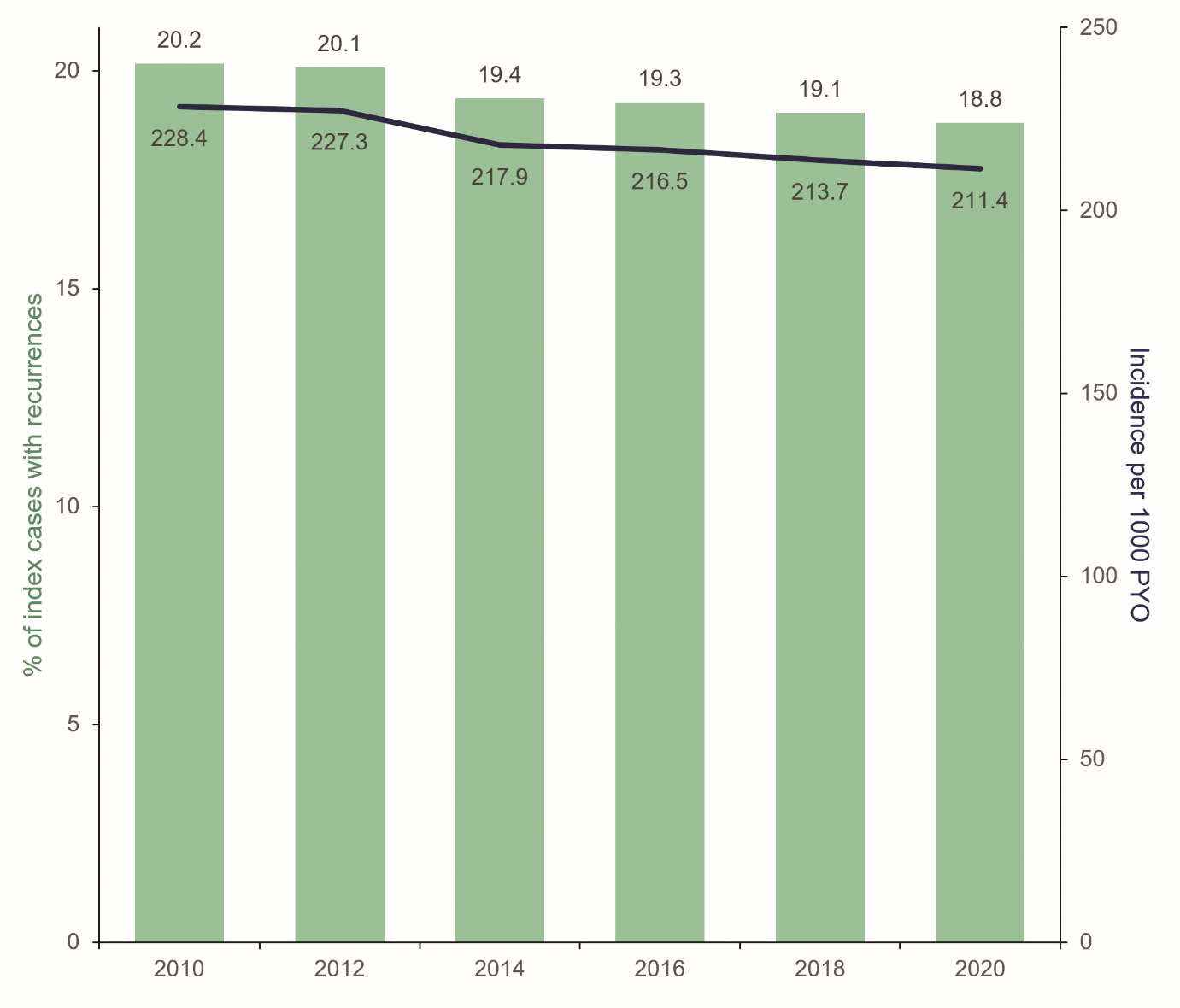
**

PYO, person-years of observation; SSTI, skin and soft tissue infection.

**Figure S2** Percentage of recurrent skin and soft tissue infections (SSTIs) by risk factors
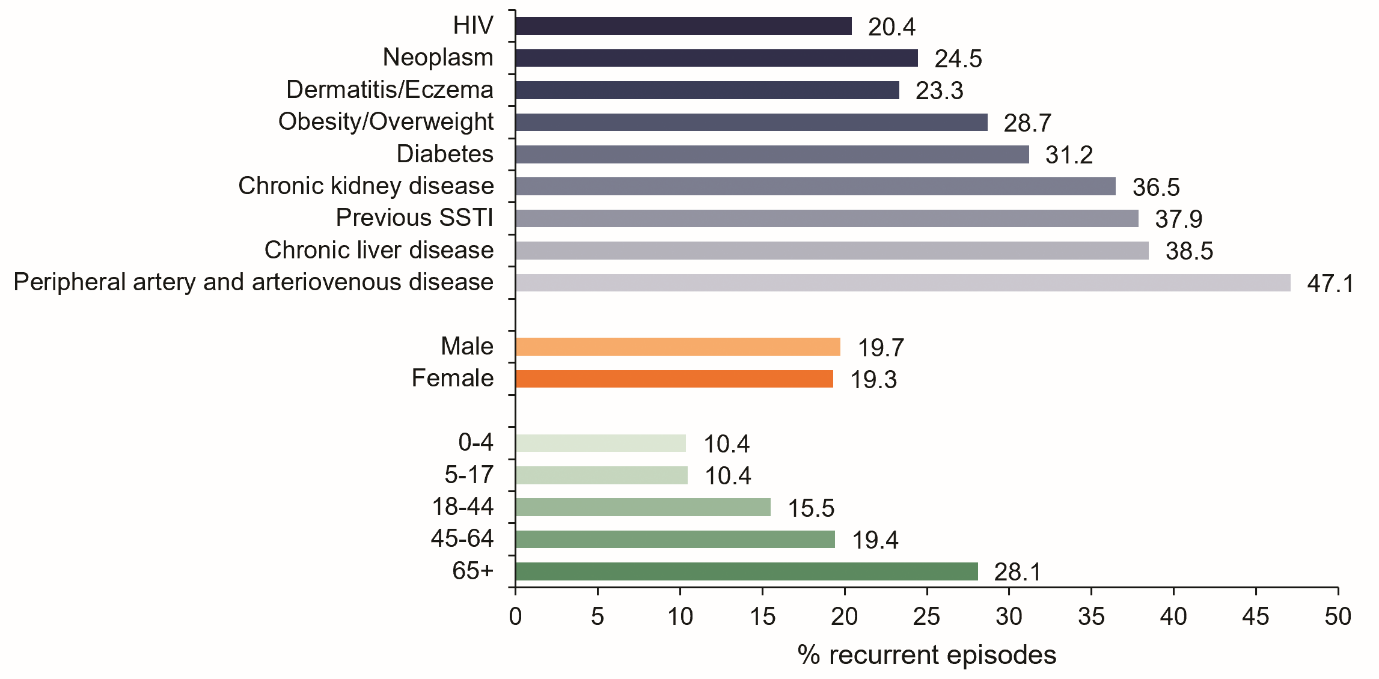


HIV, human immunodeficiency virus
