# Supplementary figures and images for: Incidence of skin and soft-tissue infections in England: 11-year retrospective study
Source: Epidemiol Infect. 2026 May 26;154:e80. doi: 10.1017/S0950268826101666 (PMC13279958; doi:10.1017/S0950268826101666)

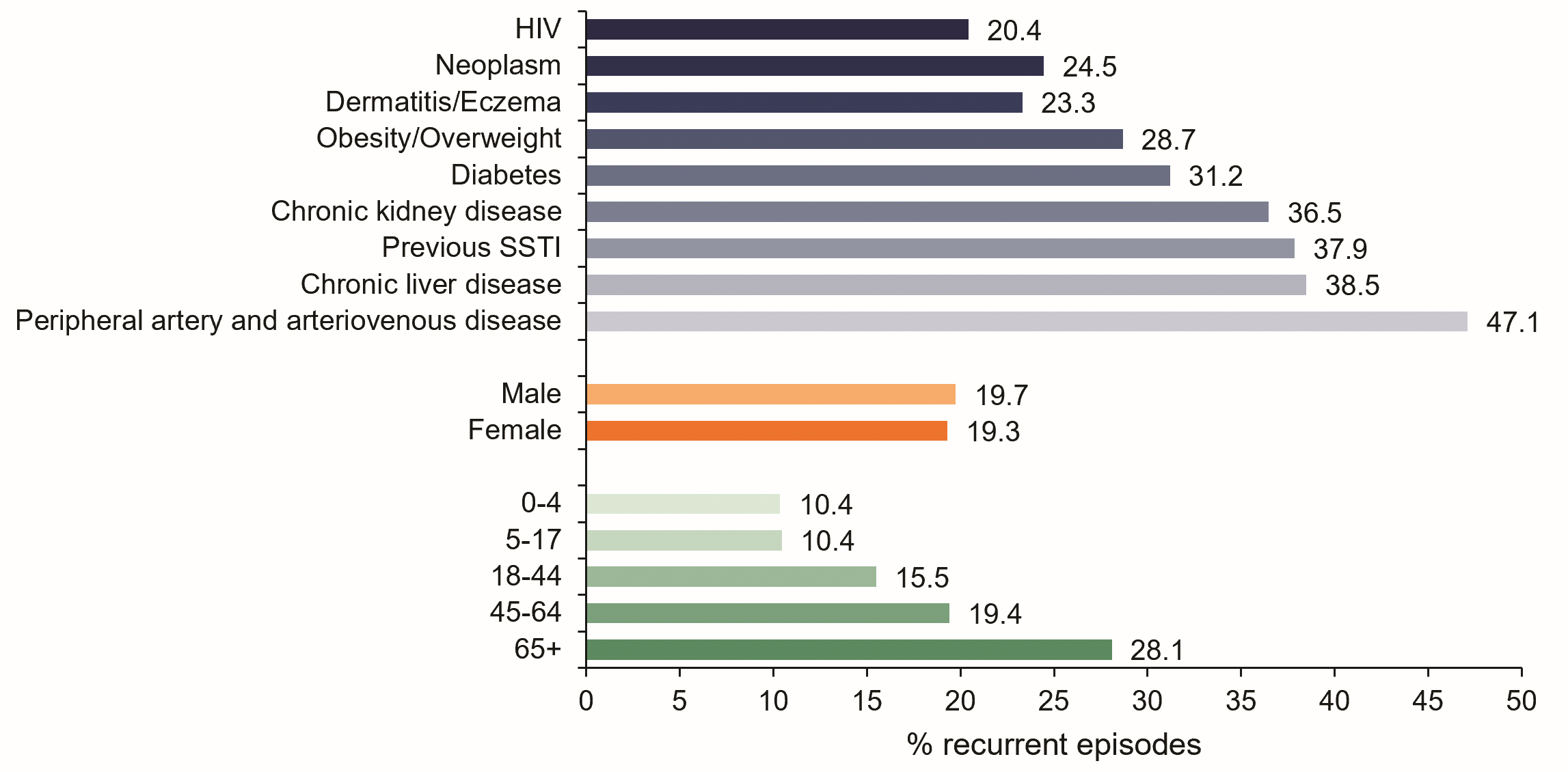

Supplement: Vella et al. supplementary material [file S0950268826101666sup001.zip › STAPH_EPI_CPRD_database study_FigureS2_CMYK.tif]

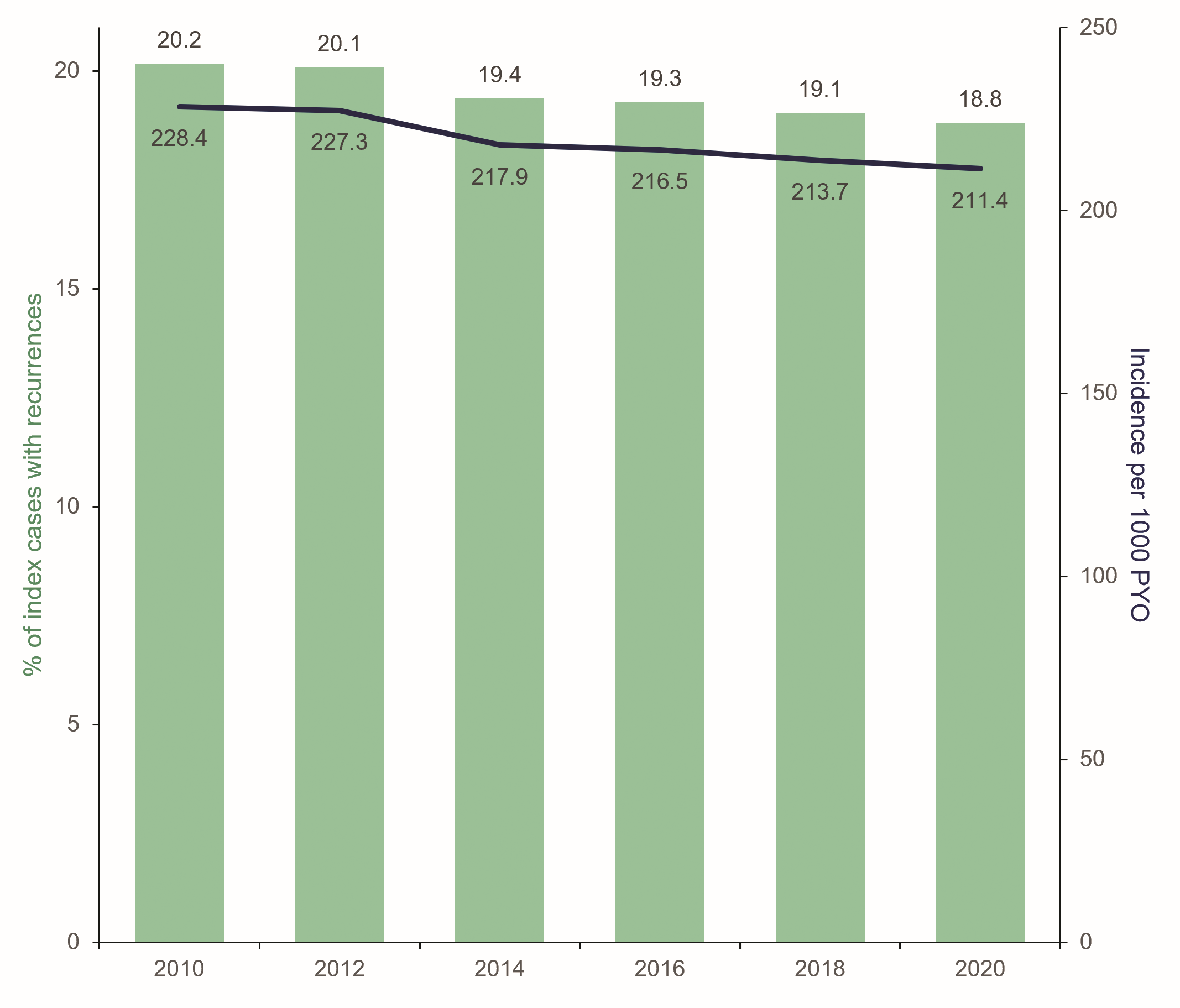

Supplement: Vella et al. supplementary material [file S0950268826101666sup001.zip › STAPH_EPI_CPRD_database study_FigureS1_CMYK.tif]
