# Supplementary material for: Incidence of skin and soft-tissue infections in England: 11-year retrospective study
Source: Epidemiol Infect. 2026 May 26;154:e80. doi: 10.1017/S0950268826101666 (PMC13279958; doi:10.1017/S0950268826101666)
Supplement: Vella et al. supplementary material [file S0950268826101666sup001.zip › STAPH_EPI_CPRD database study__Supplement material_STROBE checklist.doc]

STROBE Statement—checklist of items

|  | | Item No | Recommendation |
| --- | --- | --- | --- |
| **Title and abstract** | | 1 | (*a*) Indicate the study’s design with a commonly used term in the title or the abstract  Retrospective observational study as stated in the Abstract on page 3 |
|  |  |  | (*b*) Provide in the abstract an informative and balanced summary of what was done and what was found  Provided in Abstract on page 3 |
| Introduction | | | |
| Background/rationale | | 2 | Explain the scientific background and rationale for the investigation being reported  Included in the Introduction on pages 6 and 7 |
| Objectives | | 3 | State specific objectives, including any prespecified hypotheses  Included in the Introduction and Method on page 7 |
| Methods | | | |
| Study design | | 4 | Present key elements of study design early in the paper  Included in the Method on page 7 |
| Setting | | 5 | Describe the setting, locations, and relevant dates, including periods of recruitment, exposure, follow-up, and data collection  Included in the Method on page 7 |
| Participants | | 6 | (*a*) *Cohort study*—Give the eligibility criteria, and the sources and methods of selection of participants. Describe methods of follow-up  *Case-control study*—Give the eligibility criteria, and the sources and methods of case ascertainment and control selection. Give the rationale for the choice of cases and controls  *Cross-sectional study*—Give the eligibility criteria, and the sources and methods of selection of participants  Included in the Method on page 7-9 |
|  |  |  | (*b*) *Cohort study*—For matched studies, give matching criteria and number of exposed and unexposed  *Case-control study*—For matched studies, give matching criteria and the number of controls per case  Not applicable |
| Variables | | 7 | Clearly define all outcomes, exposures, predictors, potential confounders, and effect modifiers. Give diagnostic criteria, if applicable  Included in the Method on page 7-9 |
| Data sources/ measurement | | 8* | For each variable of interest, give sources of data and details of methods of assessment (measurement). Describe comparability of assessment methods if there is more than one group  Included in the Method on page 8-9 |
| Bias | | 9 | Describe any efforts to address potential sources of bias  Included in the Discussion on page 14-15 |
| Study size | | 10 | Explain how the study size was arrived at  Not applicable |
| Quantitative variables | | 11 | Explain how quantitative variables were handled in the analyses. If applicable, describe which groupings were chosen and why  Included in the Method on page 9 |
| Statistical methods | | 12 | (*a*) Describe all statistical methods, including those used to control for confounding  Included in the Method on page 9 |
|  |  |  | (*b*) Describe any methods used to examine subgroups and interactions  Included in the Method on page 9 |
|  |  |  | (*c*) Explain how missing data were addressed  Not applicable |
|  |  |  | (*d*) *Cohort study*—If applicable, explain how loss to follow-up was addressed  *Case-control study*—If applicable, explain how matching of cases and controls was addressed  *Cross-sectional study*—If applicable, describe analytical methods taking account of sampling strategy Not applicable |
|  |  |  | (*e*) Describe any sensitivity analyses  Not applicable |
| Results | | | |
| Participants | 13* | (a) Report numbers of individuals at each stage of study—eg numbers potentially eligible, examined for eligibility, confirmed eligible, included in the study, completing follow-up, and analysed  Included in the Results on page 10 | |
|  |  | (b) Give reasons for non-participation at each stage  Not applicable | |
|  |  | (c) Consider use of a flow diagram  Not required | |
| Descriptive data | 14* | (a) Give characteristics of study participants (eg demographic, clinical, social) and information on exposures and potential confounders  Included in the Results on page 10 | |
|  |  | (b) Indicate number of participants with missing data for each variable of interest  Not applicable | |
|  |  | (c) *Cohort study*—Summarise follow-up time (eg, average and total amount)  Not applicable | |
| Outcome data | 15* | *Cohort study*—Report numbers of outcome events or summary measures over time | |
|  |  | *Case-control study—*Report numbers in each exposure category, or summary measures of exposure | |
|  |  | *Cross-sectional study—*Report numbers of outcome events or summary measures  Included in the Results on page 10-12 | |
| Main results | 16 | (*a*) Give unadjusted estimates and, if applicable, confounder-adjusted estimates and their precision (eg, 95% confidence interval). Make clear which confounders were adjusted for and why they were included  Included in the Results on page 10-12 | |
|  |  | (*b*) Report category boundaries when continuous variables were categorized | |
|  |  | (*c*) If relevant, consider translating estimates of relative risk into absolute risk for a meaningful time period | |
| Other analyses | 17 | Report other analyses done—eg analyses of subgroups and interactions, and sensitivity analyses  Included in the Results on page 10-12 | |
| Discussion | | | |
| Key results | 18 | Summarise key results with reference to study objectives  Included in the Discussion on page 12-15 | |
| Limitations | 19 | Discuss limitations of the study, taking into account sources of potential bias or imprecision. Discuss both direction and magnitude of any potential bias  Included in the Discussion on page 14-15 | |
| Interpretation | 20 | Give a cautious overall interpretation of results considering objectives, limitations, multiplicity of analyses, results from similar studies, and other relevant evidence  Included in the Discussion on page 15 | |
| Generalisability | 21 | Discuss the generalisability (external validity) of the study results  Included in the Discussion on page 15 | |
| Other information | | | |
| Funding | 22 | Give the source of funding and the role of the funders for the present study and, if applicable, for the original study on which the present article is based  Provided in the text on pages 16 | |
